# Transcriptional responses of *Daphnia magna* exposed to Akaki river water

**DOI:** 10.1007/s10661-022-09973-y

**Published:** 2022-04-08

**Authors:** Meron Talu, Asmerom Seyoum, Berhanu Yitayew, AdaneMihret, Abraham Aseffa, Jana Jass, Gezahegne Mamo, Per-Erik Olsson

**Affiliations:** 1 The Life Science Center-Biology, School of Science and Technology, Örebro University, 701 82 Orebro, Sweden; 2 Department of Microbiology, Immunology and Veterinary Public Health, College of Veterinary Medicine and Agriculture, Addis Ababa University, Addis Ababa, Ethiopia; 3 College of Health Science Addis Ababa University, Addis Ababa, Ethiopia; 4 Armauer Hansen Research Institute (AHRI), Addis Ababa, Ethiopia

**Keywords:** Pollution, Heavy metals, Organic compounds, Waste discharge, Ecotoxicogenomics

## Abstract

Pollution of the aquatic environment is a global problem, with industrial waste, farming effluents, sewage, and wastewater as the main contributors. Many pollutants are biologically active at low concentrations, resulting in sublethal effects, which makes it a highly complex situation and difficult to assess. In many places, such as the Akaki river in Ethiopia, the pollution situation has resulted in streams with minimal presence of invertebrates or vertebrates. As it is difficult to perform a complete chemical analysis of the waters, the present study focused on using gene expression analysis as a biological end point to determine the effects of Akaki river contaminants. The present study was conducted using the small planktonic crustacean *Daphnia magna* with toxicogenomic molecular markers. *Daphnia magna* neonates were exposed to Akaki water samples collected from two different sites on the river and analyzed for mortality and expression of genes involved in different biological pathways. Despite the poor quality of Akaki river water, 48 h acute toxicity tests showed no mortality. Interestingly, analysis of sublethal toxicogenomic responses showed that exposure to Akaki water altered the expression of 25 out of 37 genes involved in metal regulation, immune response, oxidative stress, respiration, reproduction, and development. The toxicogenomic data gives insight into the mechanisms involved in causing potential adverse effects to aquatic biota harboring the Akaki river system.

**Supplementary Information:**

The online version contains supplementary material available at 10.1007/s10661-022-09973-y.

## Introduction

Contamination of water is a global problem, resulting in poor quality water that could have adverse health effects on both humans and wildlife. The rate of population growth, the expansion, and the development of new industries and other human activities contribute to pollution (APHA, 1985). Human activities include physical alterations of waters and pollution from industrial, agricultural, and residential areas (Chu & Karr, 2001), and may result in heavy metals, radioactive material, organic chemicals, and pesticide emission into waterways. This is of great concern for all living organisms (Whiles et al., 2000). Rivers running through extensively populated areas are prone to adverse impact from human activities. Improper environmental planning, lack of waste treatment facilities, and poor disposal systems are aggravating water pollution. In Ethiopia, this mainly applies to the waterways that pass through the capital.

As a developing country, Ethiopia is encountering sanitation and pollution problems. The current demographic and health surveys show that 7% of the urban population and 39% of the rural population do not have modern sanitation facilities (EDHS, 2017). Additionally, 58% of the rural population and 6% of the urban population use water from poor quality water sources (Seyoum & Graham, 2016). Based on the population census data projection for 2020, it was estimated that approximately 5 million people live in Addis Ababa (CSA, 2008), and that only 20% uses flush toilets, 74% use pit latrines, and 5.8% had no toilet facility (Beyene et al., 2015). Water contamination in Addis Ababa is aggravating due to shortage of proper sewage and waste disposal systems for the inhabitants and industries within the city. Little Akaki river is contaminated by domestic and industrial wastes, heavy metals, and nutrients (Mamo et al., 2021; Melaku et al., 2007; Weldegebriel et al., 2012).

The freshwater flea *Daphnia magna* is an important species in freshwater ecosystems and provides a link between different trophic levels (Hebert, 1978; Lampert, 2006). Their ecology and physiology are relatively well understood, and their genome is sequenced (wFleabase.org) and genetic linkage maps are available (Cristescu et al., 2006). This facilitates studies of environmental influences on gene functions that are difficult in many other invertebrate species (Colbourne et al., 2011; Eads et al., 2008). *Daphnia magna* are highly sensitive to environmental disturbances (Schindler, 1987) and can respond to stressors by changing to sexual reproduction (Hebert & Crease, 1983), the pattern of vertical migration (Dawidowicz & Loose, 1992; Stich & Lampert, 1984), and changed behavior (Gerhardt et al., 2005). Even though different studies indicate that the Akaki river is highly polluted, there is a lack of information on the toxicity of the river water to aquatic biota. Therefore, the present study aimed at using toxicogenomic analysis to determine the response of *Daphnia magna* to waters from the Akaki river and thereby shed light on the mechanisms involved.

## Materials and methods

### Description of the study area

The study area was within Addis Ababa, the capital city of Ethiopia, located at an altitude ranging from 2100 to 2700 m above sea level and covering an area of 530 km^2^ with a population of more than 5 million inhabitants. The annual average temperatures fluctuate between 10 and 25 °C (CSA, 2008). Little and Great Akaki rivers pass through the city and are used as open waste disposal sites for domestic, commercial, and industrial purposes. The wastewater volume discharged into the Akaki river is estimated to be 4.8 million m^3^ per year (CSA, 1999). The site selected for the present study was the Little Akaki river which is the western branch of the river originating from the slopes of Wechecha mountain, northwest of Addis Ababa, and flows 40 km before reaching the Aba Samuel reservoir.

### Sample collection and chemical analysis

**Fig. 1:**
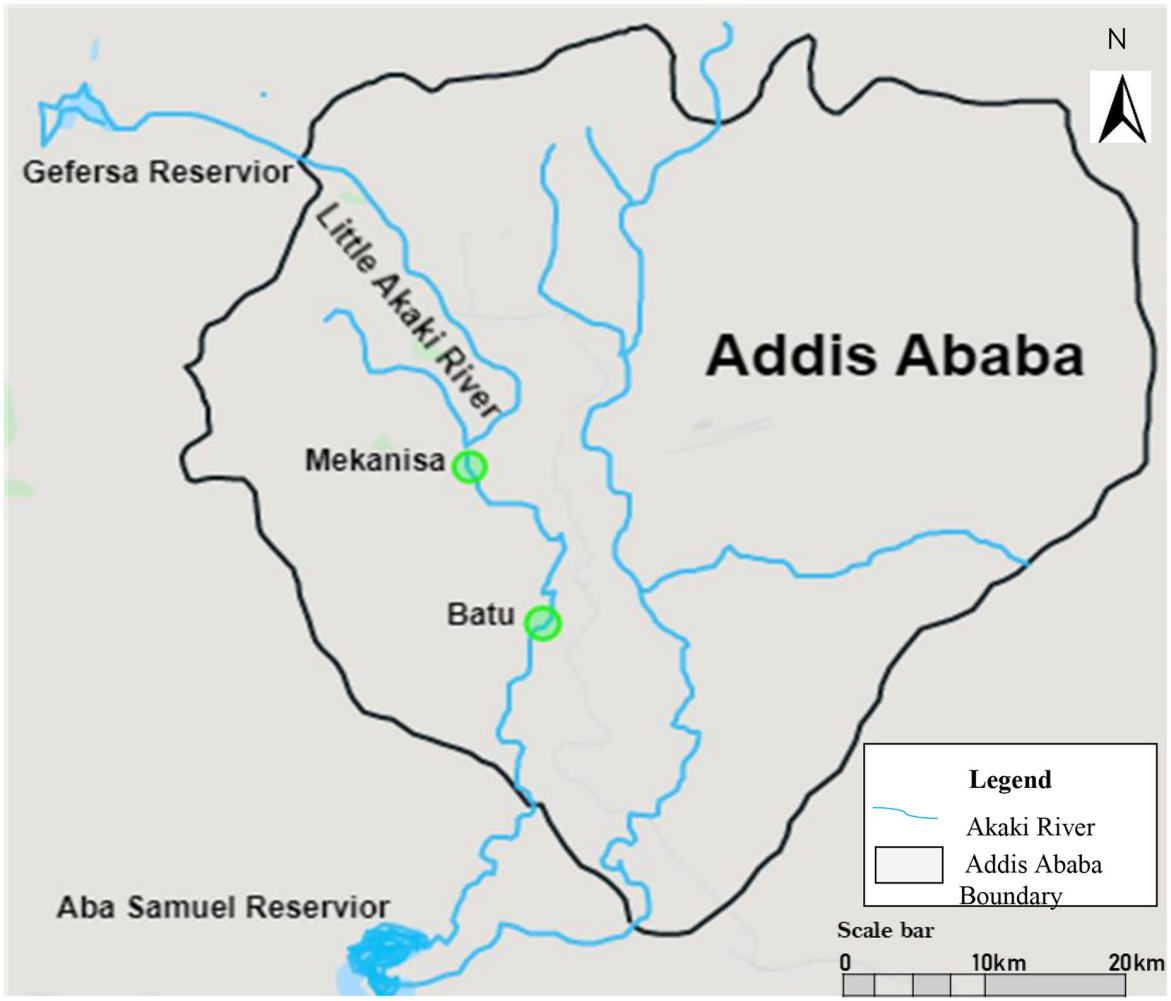
Sampling sites. The map shows Addis Ababa boundary, river network in and around Addis Ababa city, the sampling locations of Mekanisa and Batu on the Little (Tinishu) Akaki, and the downstream Lake Aba Samuel

During the dry season of the year (April 2017), water was collected for heavy metal and organic pollutant analysis as well as for gene expression analysis. Two samples were collected from site 1, Mekanisa (MK), located in the middle stream (8°58′25.7″N38°43′59.6″E), and site 2, Batu (BA), located downstream of the river (8°55′52.0″N 38°45′26.3″E) (Fig. 1). The sites were chosen based on a possible source of potentially toxic metal pollution, based on industry location, agricultural activities, and other possible sources of pollutants. Two thousand milliliters of grab water samples was collected in sterile glass containers where water temperature (°C) was measured in situ and pH measurements were done in the laboratory setting. To reduce the presence of microbes, the waters were heat-sterilized at 90 °C for 30 min and stored at 4 °C until shipment to Örebro University, Sweden, for chemical analysis and gene expression assays. Sample shipment was processed by National Soil Testing Centre under the Ministry of Agriculture and Rural Development.

Elemental analysis of the waters was performed by inductively coupled plasma quadrupole mass spectrometry (ICP-QMS; Agilent 7500cx) using external calibration solutions (Merck 10,580 multi-element standard VI). Prior to analysis, the samples were filtered (0.20 µm polypropylene) and diluted 10 times with 1% nitric acid in deionized water and 103Rh was added as an internal mass standard. Isotopes prone to di- and poly-atomic interferences, i.e., 39 K, 51 V, 53Cr, 56Fe, 63Cu, 75As, and 82Se, were quantified with the built-in Octopole Reaction System operated in collision mode with helium at a flow of 5 mL/min. DOC analysis was performed by ALS Scandinavia AB (Täby Sweden) and organic chemical analysis was performed by Eurofins AB (Lidköping, Sweden).

### Daphnia magna maintenance and exposure

As Akaki water in central Addis Ababa is void of aquatic invertebrate and vertebrate organisms the present study had to be performed as a microcosm experiment. Dormant eggs (ephippia) of the freshwater crustacean *Daphnia magna* were hatched 3 days prior to the start of exposures according to instructions in the Daphtox Kit F (Microbiotests Inc, Belgium). Briefly, one vial with ephippia was poured into a microsieve, rinsed with tap water to remove storage medium, transferred into the hatching Petri dishes in 15 mL pre-aerated standard freshwater (prepared from 67.75 mg/L NaHCO_3_, 294 mg/L CaCl_2_, 123.25 mg/L MgSO_4_, and 5.75 mg/L KCl as per manual instructions and have pH of 7.5 with a hardness of 250 mg/L CaCO_3_), and incubated for 72 h at 20 ± 1 °C under continuous light (6000 lx) and the hatching was successful. The newly hatched neonates (˂24 h old) were fed with *Spirulina microalgae* 2 h before exposure to water samples from Akaki river and reference water.

For qRT-PCR analysis, *Daphnia magna* neonates (< 24 h old) were exposed to water samples collected from BA, MK, and RW (reference water) in 5 replicates in 6-well plates for 24 h and no mortality was recorded. Twenty neonates were used for each replicate. Standard freshwater was used as RW. *Daphnia magna* were collected following exposure and the collected *Daphnia magna* were snap-frozen in liquid nitrogen and the samples were stored at − 80 °C until analyzed.

### RNA extraction and qRT-PCR analysis

*Daphnia magna* neonates (20 neonates) from each exposure group were lysed using 350 µL of Trizol reagent (Sigma), and total RNA was isolated using Direct-zol™ RNA MiniPrep Kit (Zymo Research, USA) following the manufacturer’s protocol. The RNA concentration was measured using a spectrophotometer (DeNovix, USA). One microgram of RNA was used to synthesize cDNA using qScript cDNA synthesis kit (Quanta Biosciences, USA). qRT-PCR was performed to quantify the expression of the selected 37 genes (Table S1) using qPCR BIOSyGreen Mix Lo-ROX (PCR Biosystems) using a CFX384 real-time PCR detection system (Bio-Rad, USA) with thermocycling conditions of initial denaturation step for 2 min at 95 °C, followed by 35 cycles of 95 °C for 5 s and 60 °C for 30 s. The obtained CT values were normalized using *actin* as a reference gene.

### Statistical analysis

The data was analyzed by Graph Pad Prism 8 (GraphPad Software, San Diego, USA). One-way ANOVA followed by Dennett post-test was performed to determine statistical significance, where *p* ≤ 0.05 was considered statistically significant (*p < 0.05, **p < 0.01, ***P ≤ 0.001). Ct delta was used for the analysis and the data was normalized relative to reference water (RW = 1).

Multivariate data analysis was conducted using the principal component analysis (PCA) technique to assess the variability between the samples based on gene transcriptional profile. The PCA was performed using the SIMCA software, version 13.0.3 (Umetrics, Sweden) at a significance level of 0.05, and results were presented with score plot, loading plot, dendrogram, and hierarchical cluster analysis. Values that explain the variation, R^2^X > 0.7 (goodness of fit) and Q^2^ > 0.4 (goodness of prediction) were considered to denote an acceptable model when analyzing biological data.

In order to observe whether the bioavailability of specific metals could trigger toxicity or not, the toxicity of single metals in the environmental waters was modeled using the biotic ligand model (BLM) and available water chemical parameters including pH, hardness, and dissolved organic carbon. The BLM was used to calculate the bioavailability of Cu, Pb, Ni, and Zn in the present water samples using the program, Bio-met bioavailability tool version 2.3–04-12–2013 (http://bio-met.net/).

## Results

### Chemical analysis

The results of the chemical analysis showed the concentration of most of the measured heavy metals and organic compounds was lower than the limit value set for the protection of aquatic life in freshwater (Table 1 and Table 2). The pH was 7.0 at MK and 7.6 at BA. Both water hardness and DOC levels were approximately equal at both sites (Table 2). Chemical analysis showed that the upstream site, MK, contained higher concentrations than the downstream site, BA, for most of the analyzed metals including Al, Cu, Fe, Zn, Mn, and Sr as well as for phenol, 4-n-propylphenol, and 4-ethylphenol. However, cresols and toluene were found to be higher in BA. The detected levels of p-cresol in BA and MK were 390 µg/L and 42 µg/L, respectively. Similarly, the concentration of toluene in BA and MK was 15.5 µg/L and < 1.00 µg/L, respectively (Table 2).

### Expression of stress response and immune response genes

**Fig. 2:**
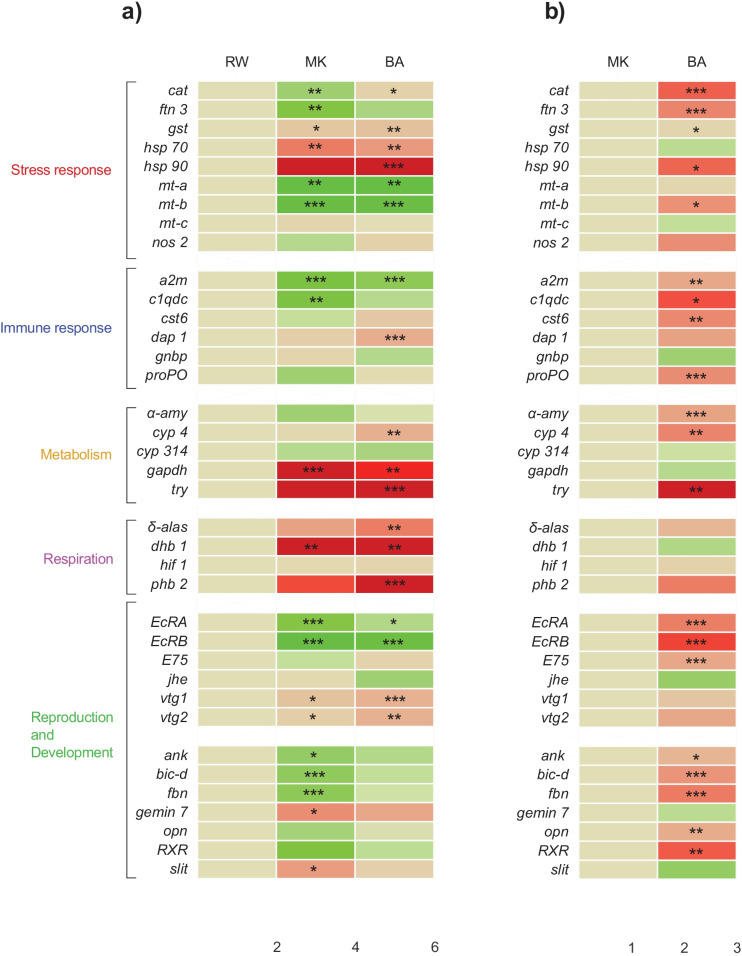
Heat map of gene expression. **a** 20 *Daphnia magna* neonates (˂ 24 h old) were exposed for 24 h to water samples from RW, MK, and BA sites and qPCR was performed. **b** Comparison of transcription level between MK and BA. The resulting data was analyzed using GraphPad prism software to generate heat map gene expression profiles. One-way ANOVA followed by Dunnett’s post-test was performed to determine statistically significant (*p ≤ 0.05, **p ≤ 0.01, ***p < 0.001) *n* = 5. The red color indicates upregulation and the green color indicates downregulation

Transcriptional analyses of stress response pathway genes including heat shock and metal response oxidative stress as well as immune response genes were analyzed. *hsp70, hsp90,* and *gst* were upregulated at both sites (Fig. 2). However, *cat* was only upregulated at the BA site and was downregulated at the MK site. In addition, two of the three *metallothionein* genes (*mt-a* and *mt-b*), typically expressed in the presence of metals, were downregulated at the BA and MK sites. *Ferritin-3* (*ftn-3*) was downregulated only at the MK site while the expression of *mt-c* and *nos2* remained unaltered (Fig. 2 and Fig. S2).

Similarly, expressions of six immune response genes, including alpha-2-macroglobulin (*a2m*), *C1q-domain-containing* (*c1qdc1*), *multicystatin6* (*cst6*), *gram-negative binding protein* (*gnbp*), *prophenol oxidase* (*propo*), and *death-associated protein 1* (*dap-1*) were analyzed*.* Of these genes, *a2m was* downregulated at both sites and *c1qdc* was downregulated only in MK (Fig. 2 and Fig. S4). The remaining immune response genes including *cst6*, *gnbp*, and *propo* remained unaltered. In addition, the apoptotic gene *dap1* was upregulated only in BA (Fig. 2 and Fig. S3).

### Expression of metabolism and respiration-related genes

Five metabolism genes including cytochromes *P450 4* and *314* (*cyp4* and *cyp314*), *α-amylase* (*α-amy*), *trypsin* (*tryp*), and *glyceraldehyde-3-phosphate dehydrogenase* (*gapdh*) were analyzed.

Both *cyp4* and *tryp* were upregulated at the BA site while no significant effects were observed at the MK site (Fig. 2 and Fig. S4). The gene *gapdh* was regulated at both the BA and MK sites, while *cyp314* and *α-amy* remained unaltered at both sites (Fig. 2 and Fig. S4).

To observe potential respiration-related effects, four respiratory genes associated with oxygen transport and mitochondrial energy consumption were analyzed. These genes include *δ-aminolevulinate synthase* (*δ-alas*), *hemoglobin-1* (*dhb1*), *hypoxia-inducible factor-1* (*hif1*), and *prohibitin2* (*phb2*). While the transcription level of *δ-alas* and *phb2* was significantly upregulated in BA, *dhb1* was significantly upregulated at both sites. In contrast, the *hypoxia-inducible factor-1* (*hif1*) remained unaltered at both sites (Fig. 2 and Fig. S5).

### Expression of reproduction and development-related genes

Out of thirteen genes associated with reproduction and development, nine were affected by the exposures. The ecdysone receptors A (*ecra*) and B (*ecrb*) were downregulated at both sites (Fig. 2 and Fig. S6). The major egg yolk proteins, *vitellogenin* (*vtg1* and *vtg2*), were upregulated at both sites (Fig. 2 and Fig. S6). The expression of genes that belong to the cytoskeleton, *ankyrin* (*ank*), and *bicaudal-d* (*bic-d*), and extracellular matrix formation including *fibrillin* (*fbn*) *gem nuclear organelle associated protein 7* (*gemin7*) and *Slit homolog* (*slit*) were analyzed. While exposure in the MK site downregulated the expression of *ank*, *bic-d*, and *fbn* and upregulated *slit* and *gemin7,* no expressional effect was observed following exposure to BA (Fig. 2 and Fig. S6). Expression of the other development-related genes including *opsin* (*opn*), *retinoid X receptor* (*rxr*), *E75 nuclear receptor* (*e75nr*), and *juvenile hormone esterase* (*jhe*) remained unaltered by both exposure sites (Fig. 2 and Fig. S6).

### Principal component analysis

**Fig. 3:**
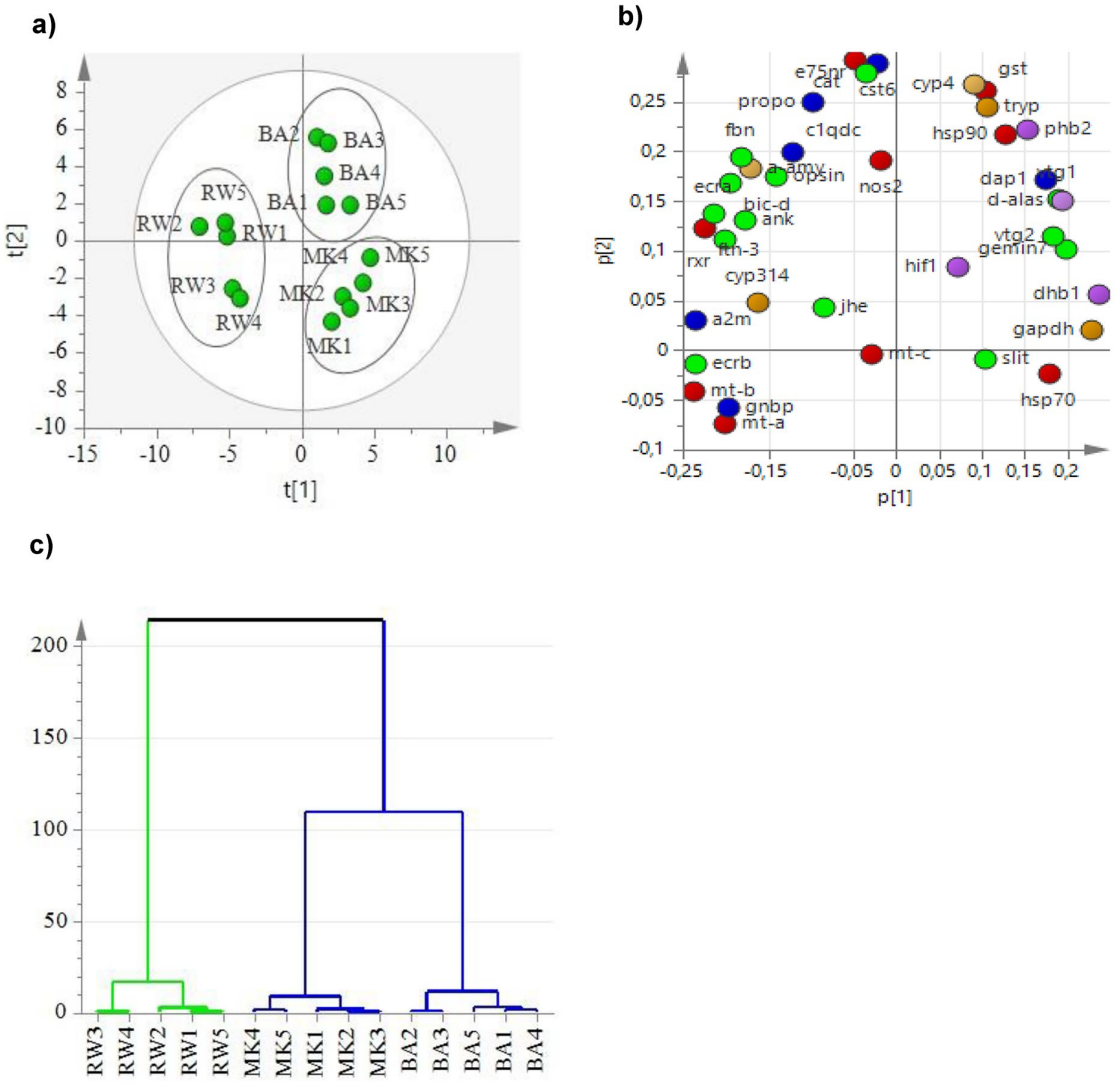
Principal component analysis and hierarchical cluster analysis of gene expression. A PCA score plot showing variance among RW and the environmental sample from Little Akaki the upstream site, MK, and downstream sites, BA, based on gene responses. **a** PCA score plot, where the first component explained 43.8% of the variance and the second component explained 27.4% of the variance. **b** PCA loading plot indicates distribution of the analyzed genes. Where the red color represents the expressed stress response genes; the blue color represents the immune response genes; the yellow color represents the metabolism genes; the purple color indicates the expressed respiration genes; and the green color indicates the expressed reproduction and development-related genes. **c** PCA dendrogram showing the connectivity of clusters generated by cluster analysis. The green line indicates the group 1 cluster, i.e., RW, and the blue line indicates the group 2 clusters of environmental water samples from MK and BA

A multivariate PCA analysis was performed to analyze variance among treatments (RW, MK, and BA) based on the change in the expression of the 37 genes analyzed in exposed *Daphnia magna*. The analysis provided a goodness of fit (R^2^X) value of 0.713 and goodness of prediction (Q^2^X) value of 0.544. Analysis of the score plot (Fig. 3a) showed that principal component 1 (PC1) explained 43.8% of the variation, while the principal component 2 (PC2) explained 27.4% of the variation. To show the distribution of variable PCA, a loading plot (Fig. 3b) was generated. The data shows that PC1 explained the distinct difference in gene expression between RW and the environmental waters (MK and the BA). PC2 shows distinct variation among the environmental samples MK and BA. This was further confirmed from the dendrogram of cluster analysis (Fig. 3c) where the environmental water samples MK and BA formed two separate groups and separated from the reference water cluster. Transcription level differences between the two sampling sites are shown in Fig. 2b.

### Biotic ligand model

The biotic ligand model (BLM) was used for individual metals (e.g., Pb, Cu, Zn, Ni). This model aims to predict local “predicted no-effect concentration” (PNEC) values taking the site-specific physico-chemical water conditions into account, hence considering bioavailability in its toxicity estimations. Using the Bio-met tool and river Akaki site-specific pH (7–7.6), DOC (33.2–32.7 mg/L), and Ca (81–70 mg/L), local EQS-based PNEC values were calculated: 0.18–0.03 ug/L for Cu, 1.37–0.42 ug/L for Zn, 0.44–0.49 ug/L for Ni, and 0.02–0 ug/L for Pb. The calculated EQS values indicated that the bioavailability of selected metals was not a contributor to the toxicity. In addition, the BLM risk characterization ratio (RCR) was 0.18–0.03 for Cu, 0.11–012 for Ni, 0.13–0.04 for Zn, and 0.02–0 for Pb, indicating that no risk present from this metal’s exposure.

## Discussion

Except for iron, which exceeded the maximum recommended level set by the US Environmental Protection Agency (USEPA) for freshwater (1 mg/L) at site MK, the concentrations of other heavy metals did not exceed the maximum concentration set for the protection of aquatic life in freshwater (USEPA, 2015, CCME, 1991).

The elevated transcription of the stress response genes (*hsp70*, *hsp90*, *gst*, and *cat*) indicates increased levels of oxidative stress, which could be associated with the presence of the phenolic compounds at both sites. In a recent study, it was observed that exposure of endothelial cells and U937 cells to p-cresol resulted in ROS production (Chang et al., 2014) and that exposure of mice to phenol caused oxidative stress in the skin (Murray et al., 2007). Metabolism of phenol has also been shown to result in the formation of phenoxyl radicals that cause oxidative stress and damage to proteins, DNA, and lipids (Stoyanovosky et al., 1996). Thus, the detected phenolic compounds in Akaki water may contribute to the observed toxicogenomic oxidative stress response.

Two of the three *mt* genes and *ftn-3* were downregulated. Downregulation of *mt* has been observed in other environmental studies (Ghazy et al., 2017; Kumar et al., 2015; Seyoum & Pradhan, 2019; Thummabancha et al., 2016). Studies on *Daphnia magna* have shown that heavy metals such as cadmium (Li et al., 2016), zinc (Fan et al., 2009), titanium dioxide (Tan & Wang, 2014), and mercury (Tsui & Wang, 2005) induced *mt* expression in a concentration-dependent manner. In Nile tilapia from Lake Burullus in northern Egypt, it was observed that the *mt* gene expression was downregulated even though the lake had relatively high metal levels (Ghazy et al., 2017). In another study, it was observed that exposure of human HepG2 cells to clofibric acid resulted in inhibition of *mt* (Bianchi et al., 2002). Furthermore, co-exposure of *Corbicula fluminea*, a freshwater clam, to Cd and an organophosphate flame retardant mixture resulted in downregulation of *mt* gene transcription when compared to Cd exposure alone (Li et al., 2018). Thus, there is emerging data indicating that *mt* may be downregulated in the environment in the presence of xenobiotics, which ultimately can render organisms more susceptible to metal toxicity. This suggests that even though the level of most heavy metals in the Akaki river is below the permissible limit, they may still cause toxicity to *Daphnia magna* due to the observed inhibition of *mt* transcription.

The immune response genes *a2m* and *c1qdc* as well as the apoptotic gene *dap-1* were altered following exposure to Akaki waters. An increased expression of *a2m*, a protein that inhibits pathogen proteases, is associated with increased stimulation of subsequent immune signal transduction pathways that promote the defense against microbial infections (Jin et al., 2012; Little et al., 2004; Ponprateep et al., 2017). Together with stress responses, it maintains cellular homeostasis (Chovatiya & Medzhitov, 2014). However, when the stress level becomes too severe, the cells start to die through the activation of apoptotic pathways (Berridge, 2012). Inhibition of the immune response genes *a2m* and *c1qdc* suggests increased susceptibility to microbial infection (Janeway & Medzhitov, 2002; Jin et al., 2012; Li et al., 2019). Therefore, the observed downregulation of *a2m*and *c1qdc*, along with the upregulation of *dap-1* suggests that the *Daphnia magna* may become more susceptible to bacterial infection by Akaki river water.

The metabolism genes *cyp4*, *tryp*, and *gpdh* were altered following exposure to Akaki waters. Upregulation of *Cyp4* at the BA site may be related to the high levels of p-cresol (390 µg/L). It can be noted that humans normally excrete approximately 50 mg of p-cresol in the urine daily (Sullivan & Krieger, 1999) thus contributing to the observed p-cresol levels in the Akaki river. A study in *Daphnia magna* has shown that *trypsin* expression is regulated by the type of ingested food. Trypsin has been shown to be upregulated after *Daphnia magna* were fed a diet of 20% blue-green algae or cyanobacteria containing food (Perera et al., 2012; von Elert et al., 2004). The Akaki river receives nutrient input from sewage and waste disposal from inhabitants and industry and this may contribute to the blooming of cyanobacteria which ultimately could cause upregulation of *tryp* at the RNA level. Induction of *gapdh expression* indicates a stress response that may lead to cell death (Tristan et al., 2011). Thus, the observed upregulation of *gapdh* in both BA and MK could be due to activation of innate immunity and apoptotic pathways.

Changes in the expression of the cytoskeleton (*ank* and *bic-d*) and extracellular matrix (*fbn, germin7,* and *slit*) genes that are needed for structural frameworks and cell organization (Burger et al., 2019; Muncie & Weaver, 2018) by MK water may lead to cytoskeleton instability and disruption of cellular functions. These effects may be related to the higher chemical level measured at MK than at the downstream site BA. The absence of these effects on BA sites also indicates the presence of site-specific toxicity in the Akaki river.

The altered transcription of e*cra*, *ecrb*, *vtg1*, and *vtg2* both by BA and MK suggests that the Little Akaki river water interferes with *Daphnia magna* reproduction and development. *VTG* transcription is an established biomarker to assess effects on reproduction (Hannas et al., 2011; Jeong et al., 2013).

Respiration genes (*dhb1, phb-2,* and *δ-alas*) were also altered following exposure of *Daphnia magna* to the Akaki water. In other studies, exposure of *Daphnia magna* to organic compounds, including atrazine and the flame retardant tris(2-butoxyethyl)phosphate resulted in upregulation of *dhb1* (Giraudo et al., 2015; Rider & LeBlanc, 2006). The observed upregulation of *dhb1* may be related to the presence of organic pollutants in the river. The alteration in the transcription of *δ-alas* genes which is involved in heme biosynthesis (Brown et al., 2018; Vandenbrouck et al., 2009) and *phb-2*, which takes part in oxygen transportation and mitochondrial respiration (Bavelloni et al., 2015; Signorile et al., 2019), suggests that the Akaki river water would cause respiratory effects on *Daphnia magna* (Fig. 2 and Fig. S6).

## Conclusions

In the present study, we evaluated the toxic effect of the Little Akaki river water using gene transcription analysis. The results demonstrate that the water disrupts multiple functions, including induction in oxidative stress, inhibition of metal toxicity and immune response genes, alterations of the expression of genes involved in respiration, reproduction, and developmental pathways. In addition, we show that there are site-specific differences in the gene expression profiles from the two sampling sites. This demonstrates the importance of site-specific analysis to determine the nature of the toxicity of environmental pollution. The present study gives insight into the toxicity of the Akaki river water indicating multiple regulatory dysfunctions ultimately contributing to the lack of aquatic life living in the river system.

## Data sets

All data generated or analyzed during this study are included in this published article (and its supplementary information files).

**Table 1:** Metal composition of Akaki river water

	LOQ**		**Site MK**				**Site BA**		
**element**	**µg/L**		**mg/L**	**SD**	**RSD (%)**		**mg/L**	**SD**	**RSD (%)**
K*	18.40		123.86	2.44	1.97		60.30	1.53	2.53
Na	8.50		98.31	0.07	0.07		121.96	1.25	1.02
Ca	5.00		80.96	0.39	0.48		69.51	0.10	0.15
Mg	0.05		21.03	0.35	1.67		20.02	0.28	1.41
Mn	0.10		2.09	0.02	0.78		1.31	0.00	0.11
**element**	**µg/L**		**µg/L**	**SD**	**RSD (%)**		**µg/L**	**SD**	**RSD (%)**
Fe*	0.10		1768.20	13.98	0.79		348.64	3.45	0.99
Sr	0.05		395.99	1.60	0.40		353.62	1.73	0.49
Ba	0.05		169.90	0.51	0.30		79.47	0.61	0.77
Rb	0.05		53.44	0.45	0.84		36.68	0.35	0.96
Al	0.10		27.23	1.35	4.95		18.84	0.30	1.58
Cu*	0.05		17.91	2.30	12.83		1.55	0.00	0.02
Zn	0.10		12.06	0.37	3.07		6.29	0.20	3.23
Ga	0.05		6.42	0.12	1.88		3.02	0.05	1.71
Ni	0.10		5.36	0.17	3.20		5.52	0.25	4.47
V*	0.05		4.99	0.07	1.33		3.88	0.06	1.56
Cr*	0.20		4.00	0.06	1.54		4.81	0.15	3.13
Se*	1.30		3.61	0.41	11.42		5.06	2.49	49.24
Mo	0.05		3.33	0.20	6.01		3.73	0.14	3.85
Li	0.10		2.49	0.09	3.76		6.08	0.15	2.42
U	0.05		1.43	0.15	10.41		0.88	0.02	1.85
Co	0.05		1.37	0.08	5.60		1.47	0.04	2.42
As*	0.10		1.15	0.11	9.37		1.44	0.19	13.42
Pb	0.05		1.01	0.09	8.64		0.24	0.00	1.04
Te	0.30		<0.30	-	-		<0.30	-	-
Bi	0.05		0.23	0.03	14.88		0.14	0.01	5.50
Be	0.05		0.08	0.01	17.01		0.05	0.05	94.83
Ag	0.05		<0.05	-	-		<0.05	-	-
Cd	0.05		<0.05	-	-		<0.05	-	-
Tl	0.05		<0.05	-	-		<0.05	-	-
samples									

*Analysis using collision cell to avoid impact from di- and polyatomic interferences.

**Limit of quantification. Note the µg/L for LOQ and mg/L for samples. *SD* standard deviation.

*RSD* relative standard deviation = SD/mean*100

**Table 2:**
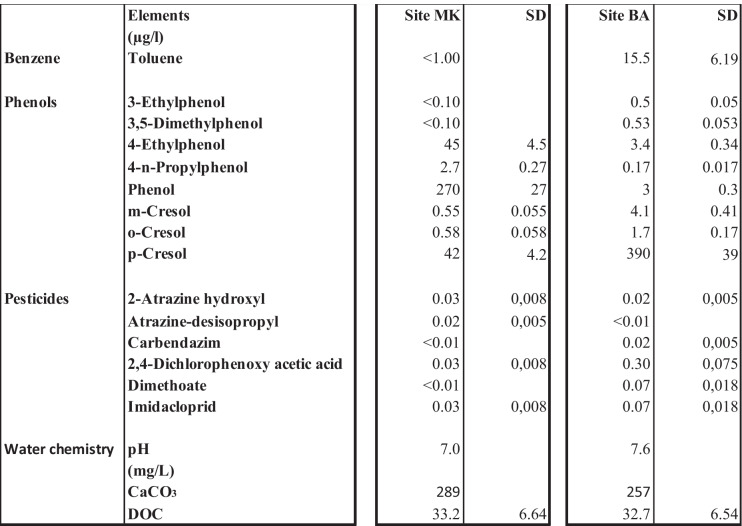
Organic compounds and water chemistry

## Supplementary Information

Below is the link to the electronic supplementary material.

Supplementary file1 (PDF 151 KB)

Supplementary file2 (DOCX 120 KB)

## Data Availability

Not applicable.
