# Supplementary material for: Transcriptional responses of Daphnia magna exposed to Akaki river water
Source: Environ Monit Assess. 2022 Apr 8;194(5):349. doi: 10.1007/s10661-022-09973-y (PMC8993723; doi:10.1007/s10661-022-09973-y)
Supplement: Supplementary file 1 — Supplementary file1 (PDF 151 KB) [file 10661_2022_9973_MOESM1_ESM.pdf]

Fig. S1: Molecular structure of organic chemicals detected in Akaki river

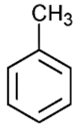
Toluene

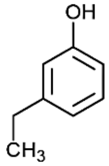
3-Ethylphenol

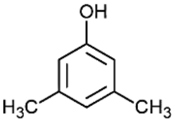
3,5-Dimethylphenol

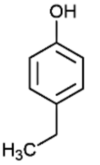
4-Ethylphenol

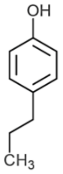
4-n-Propylphenol

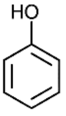
Phenol

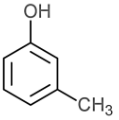
m-Cresol

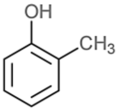
o-Cresol

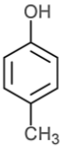
p-cresol

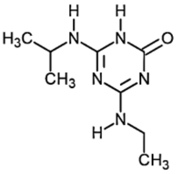
2-Atrazine hydroxyl

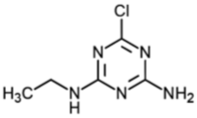
Atrazine-desisopropyl

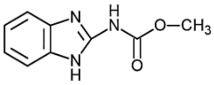
Carbendazim

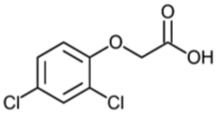
2,4-dichlorophenoxy

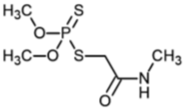
Dimethoate

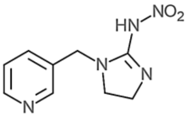
Imidacloprid

Fig. S2

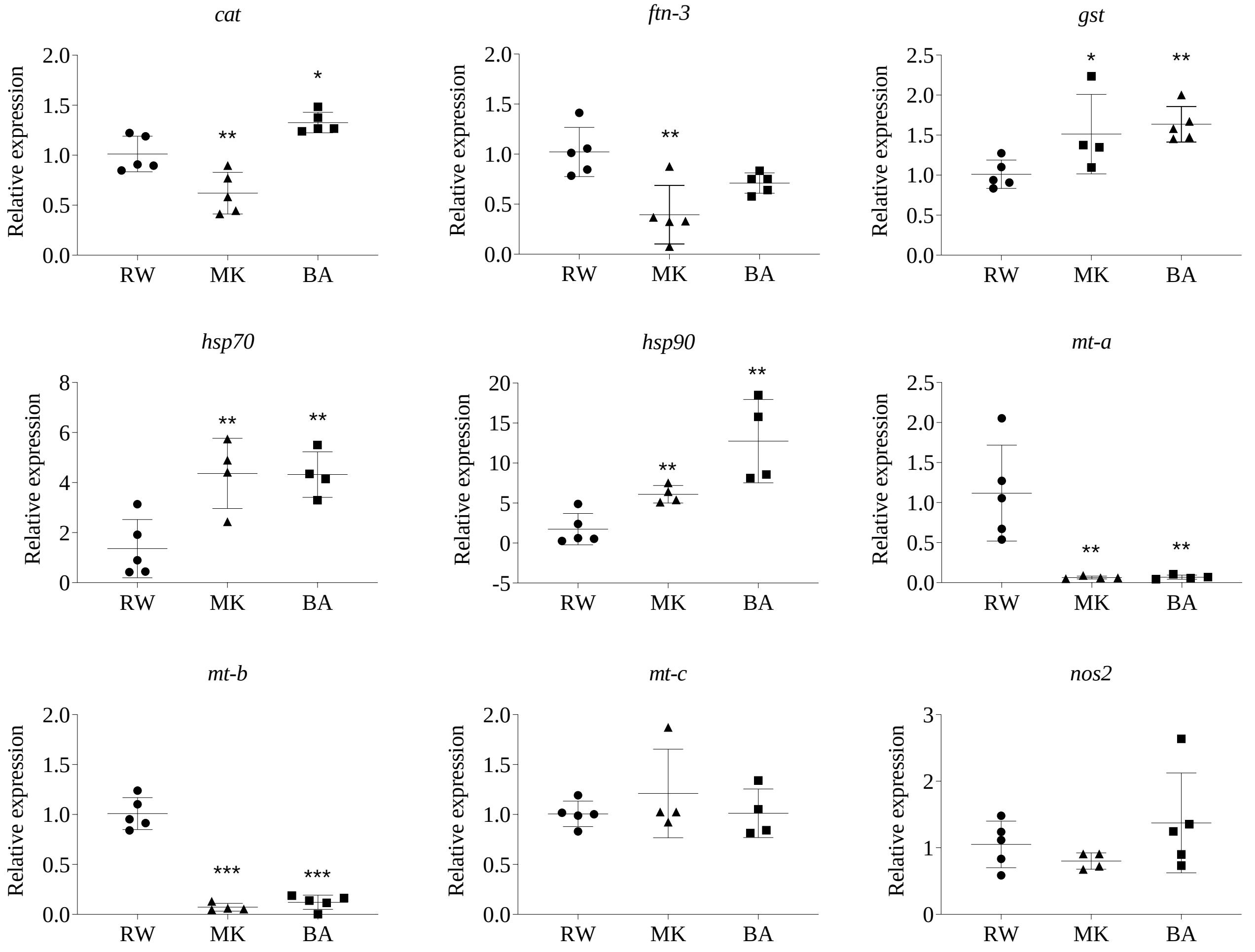

cat
ftn-3
gst
hsp70
hsp90
mt-a
mt-b
mt-c
nos2
Relative expression
RW
MK
BA

Fig. S3

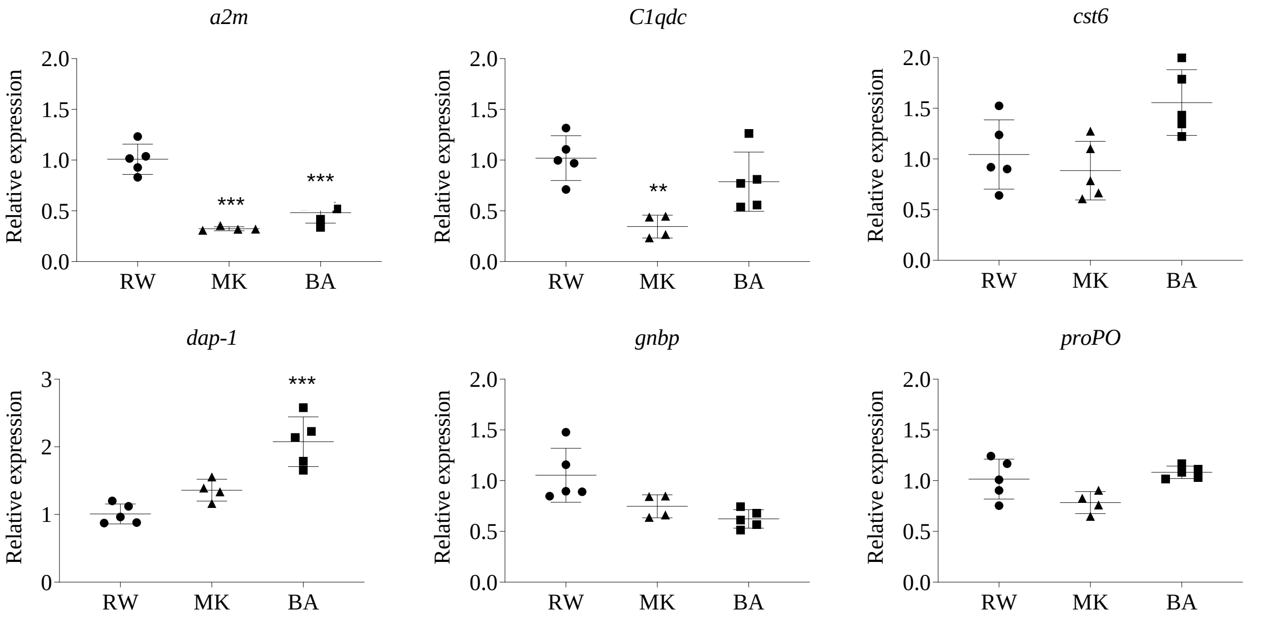

a2m
C1qdc
cst6
Relative expression
2.0
1.5
1.0
0.5
0.0
***
***
**
RW
MK
BA
dap-1
gnbp
proPO
3
2
1
0
***

Fig. S4

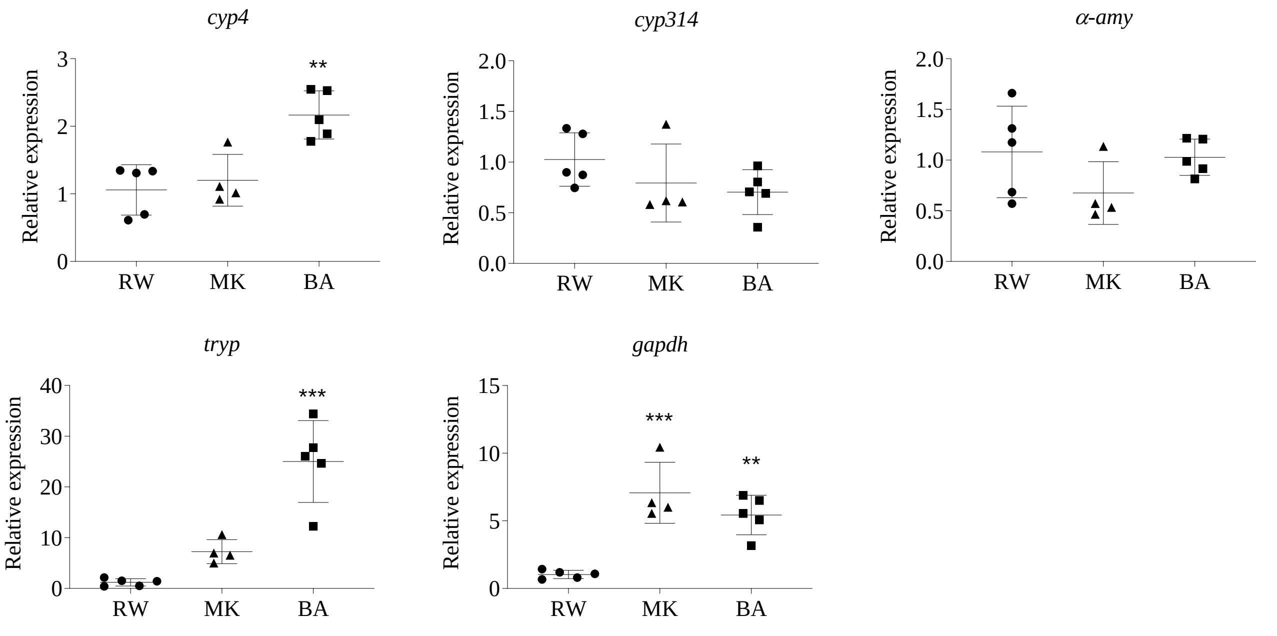
cyp4
cyp314
α-amy
tryp
gapdh
Relative expression
RW
MK
BA
**
***
***
**
0
1
2
3
0.0
0.5
1.0
1.5
2.0
0
10
20
30
40
0
5
10
15

Fig. S5

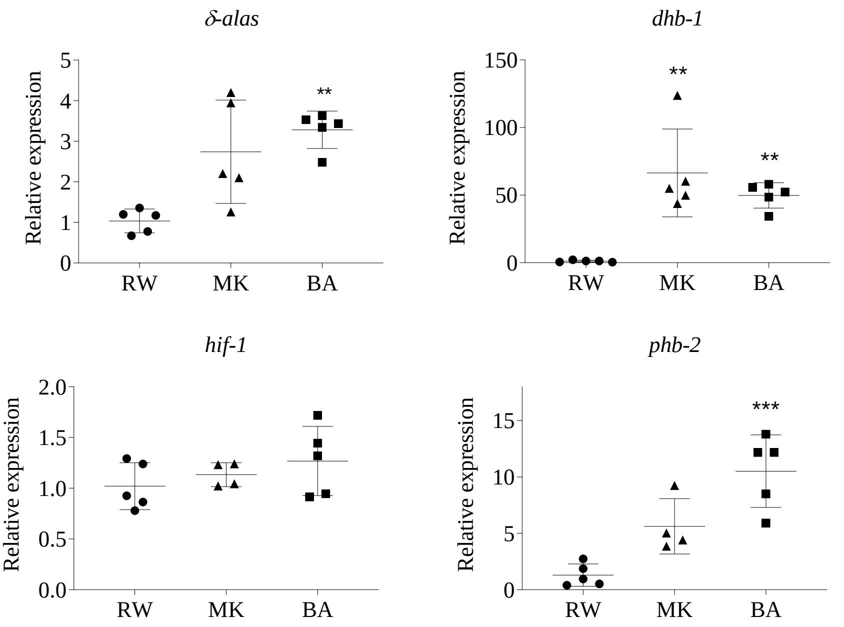

δ-alas
dhb-1
hif-1
phb-2
Relative expression
RW
MK
BA
**
***
0
1
2
3
4
5
50
100
150
0.0
0.5
1.0
1.5
2.0
10
15

Fig. S6

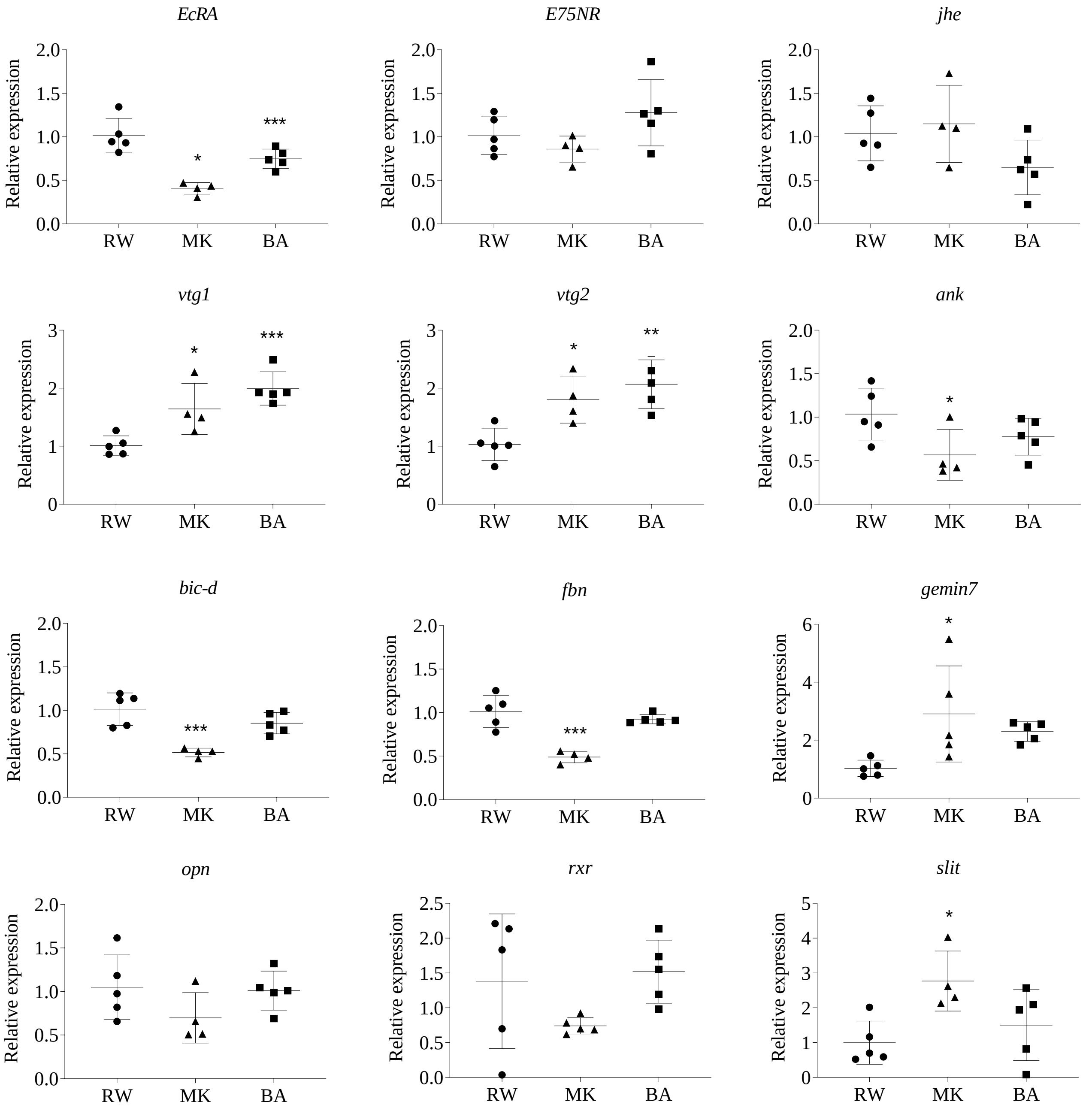
EcRA
E75NR
jhe
vtg1
vtg2
ank
bic-d
fbn
gemin7
opn
rxr
slit
Relative expression
RW
MK
BA
*
**
***
