# Supplementary material for: Transcriptional responses of Daphnia magna exposed to Akaki river water
Source: Environ Monit Assess. 2022 Apr 8;194(5):349. doi: 10.1007/s10661-022-09973-y (PMC8993723; doi:10.1007/s10661-022-09973-y)
Supplement: Supplementary file 2 — Supplementary file2 (DOCX 120 KB) [file 10661_2022_9973_MOESM2_ESM.docx]

**Supplementary information**

Table S1. Primers used for qRT-PCR analysis
